# Editorial: Food Biopreservation Technologies: Current Trends and Approaches

**DOI:** 10.3389/fmicb.2022.907198

**Published:** 2022-04-26

**Authors:** Anthoula A. Argyri, Agapi I. Doulgeraki, Chrysoula C. Tassou, Jörg Hummerjohann

**Affiliations:** ^1^Institute of Technology of Agricultural Products, Hellenic Agricultural Organization-DIMITRA, Lycovrissi, Greece; ^2^Agroscope, Zurich, Switzerland

**Keywords:** biopreservation, protective cultures, multi-functional cultures, bioprotective agents, biocontrol agents, antimicrobial metabolites

Biopreservation is defined as the use of microorganisms and/or their metabolic products to extend the shelf life and enhance the safety of foods. The aim of this Research Topic was to present novel approaches and findings in the field of bioprotective microorganisms and multi-functional cultures. These approaches included a) novel microbial cultures or culture's metabolites that enhance food safety and quality, tested in real food ecosystems (*in situ*), b) exploitation of the mechanisms behind the antimicrobial activity *in vitro* and *in situ*–bio-chemical and/or molecular targeted genes analysis and c) novel analytical methods that provide new insights in the production, processing and preservation of the novel food products.

Lactic acid bacteria (LAB) have been the most studied biopreservation agents, contributing as protective cultures in food safety and/or shelf life of the products. However, there is a growing interest in multi-functional cultures (i.e., starter/adjunct/probiotic/protective cultures) that may additionally enhance the organoleptic and physicochemical characteristics (technological properties) and/or improve the functional properties (probiotic and prebiotic potential) of foods. Within this topic, 2 research articles have elaborated with the antimicrobial properties of bioprotective/functional LAB isolates against spoilage or pathogenic microbiota that are of major concern in dairy products (Shi and Knøchel; Papadopoulou et al.). The other 2 articles dealt with the effectiveness of the antifungal extract produced by a *Streptomyces* strain on controlling citrus postharvest green mold (Lin et al.) and the application value of a cold-active glucose oxidase isolated from *Penicillium* and produced heterologously in *Pichia* as a grass carp biopreservative (Yuan et al.).

As mold spoilage is a major concern in dairy industry, Shi and Knøchel have studied the antimicrobial potential of 12 LAB isolates (including strains of *Lacticaseibacillus rhamnosus, Lactiplantibacillus plantarum, Lentilactobacillus parabuchneri, Lacticaseibacillus paracasei*) against four *Mucor* and nine *Penicillium* strains, isolated from dairy products. Different inactivation patterns were observed depending on the growth medium studied. Strong fungal inhibition was observed when MRS was used as a growth medium, but a reduced LAB growth and a weaker inhibition of mold growth were observed when yogurt serum medium was employed. Removal of the microbial cells caused almost complete loss of activity, however it was noted that combining cultures could in some cases enhance the inhibition efficacy. Furthermore, the manganese depletion by *L. plantarum* LP37 was found to play an important role in the growth inhibition of both the *Penicillium* and the *Mucor* strains tested in yogurt. The authors have concluded that apart from a non-affected *Penicillium roqueforti* strain (ISI4), the tested spoilage molds were generally sensitive to bioprotective cultures capable of making the manganese unavailable in the matrix, indicating possible expansion of their application in similar matrices.

Papadopoulou et al. explored the performance of a LAB strain with probiotic potential as an adjunct culture, in parallel with the typical yogurt starters, to produce a new functional product with increased safety. The findings of the study demonstrated that the potential probiotic *L. plantarum* T571 strain added in yogurt was viable and in adequate population levels for conferring a possible health benefit to the consumer, throughout the yogurt storage at 4 and 12°C. Moreover, the addition of this strain led to quality products with desirable organoleptic characteristics. The *in situ* antimicrobial activity of *L. plantarum* T571 strain was also tested in yogurts artificially contaminated with a cocktail mixture of three strains of *Listeria monocytogenes* in two different initial levels of inoculum. It was shown that the addition of the *L. plantarum* T571 strain led to reduced population of *L. monocytogenes* in a shorter time period compared to the control, at both storage temperatures. Furthermore, in this study, Fourier transform infrared spectroscopy in combination with partial least squares and support vector machine regression and classification models was shown to be a promising rapid and noninvasive method to estimate the microbial counts (Total viable counts, LAB, and lactococci/*Streptococcus thermophilus*) and the sensorial characteristics of yogurt samples throughout their shelf life.

In another approach, *Streptomyces* was evaluated for the possible prevention and control of postharvest fruit diseases (Lin et al.). The authors have explored the effectiveness of the antifungal extract produced by *Streptomyces lavendulae* X33 against *Penicillium digitatum*, a fungus that causes severe fruit decay symptoms on infected citrus fruit. The study aimed to identify differentially expressed proteins in *P. digitatum* stimulated by active substances via isobaric tags through relative and absolute quantitation (iTRAQ) approach and explore the intrinsic molecular mechanism of the X33 antifungal extract on *P. digitatum*. It was shown that the X33 extract induced mitochondrial membrane dysfunction and cellular integrity impairment, which subsequently can affect energy metabolism, oxidative stress, and transmembrane transport. The fact that the oxidative stress of *P. digitatum* was induced by the X33 extract was justified through the improved alkaline phosphatase activity, extracellular conductivity, increased H_2_O_2_ and malondialdehyde contents, inhibition of energy, amino acid, and sugar metabolism. The findings of the study suggested that the *Str. lavendulae* X33 antifungal extract may be an effective fungicide for controlling citrus postharvest green mold.

Yuan et al. proposed a glucose oxidase (GOx) as a biopreservative with great application potential in food preservation. However, the GOx must show high enzyme activity at low temperature (4°C) to be useful for aquatic products preservation. In their study, Yuan et al. (2020) isolated a new cold-active GOx from *Penicillium* sp. MX3343 and successfully expressed heterologously in *Pichia pastoris* X33. The biochemical and antimicrobial characteristics of recombinant enzymes were analyzed in depth to prove the significant effects on the preservation of grass carp. It was shown that, the recombinant enzyme named GOxP5 was able to maintain the 72% of maximum activity at 4°C and was stable at a broad pH range from pH 2–6, suggesting its potential application for the aquatic products cold preservation. The sensory, microbiological (total bacterial count) and physicochemical (total volatile basic nitrogen and pH) analyses during fish filets cold storage (4°C), proved that GOxP5 can be an excellent freshness preserving agent. In addition, GOxP5 presented good antimicrobial activity against *L. monocytogenes* and *Vibrio parahaemolyticus*, representing two common fish pathogenic bacteria. The authors have concluded that the cold-active GOxP5 has great application value as a grass carp biopreservative and its application could be further explored along with other preservatives.

In conclusion, the articles included in this Research Topic provided several examples of biopreservation agents (microorganisms or their metabolites) to improve the shelf life and/or safety of dairy, fruit and fish products.

## Author Contributions

All authors listed have made a substantial, direct, and intellectual contribution to the work and approved it for publication.

## Conflict of Interest

The authors declare that the research was conducted in the absence of any commercial or financial relationships that could be construed as a potential conflict of interest.

## Publisher's Note

All claims expressed in this article are solely those of the authors and do not necessarily represent those of their affiliated organizations, or those of the publisher, the editors and the reviewers. Any product that may be evaluated in this article, or claim that may be made by its manufacturer, is not guaranteed or endorsed by the publisher.

